# Clinical commissioning of an adaptive radiotherapy platform: Results and recommendations

**DOI:** 10.1002/acm2.13801

**Published:** 2022-10-31

**Authors:** Kelly Kisling, Timothy D. Keiper, Daniela Branco, Grace Gwe‐Ya Kim, Kevin L Moore, Xenia Ray

**Affiliations:** ^1^ Department of Radiation Medicine and Applied Sciences University of California San Diego San Diego California USA

**Keywords:** adaptive radiotherapy, CBCT, ethos, on‐table adaptation, quality assurance, synthetic CT, workflow

## Abstract

Online adaptive radiotherapy platforms present a unique challenge for commissioning as guidance is lacking and specialized adaptive equipment, such as deformable phantoms, are rare. We designed a novel adaptive commissioning process consisting of end‐to‐end tests using standard clinical resources. These tests were designed to simulate anatomical changes regularly observed at patient treatments. The test results will inform users of the magnitude of uncertainty from on‐treatment changes during the adaptive workflow and the limitations of their systems. We implemented these tests for the cone‐beam computed tomography (CT)‐based Varian Ethos online adaptive platform.

Many adaptive platforms perform online dose calculation on a synthetic CT (synCT). To assess the impact of the synCT generation and online dose calculation on dosimetric accuracy, we conducted end‐to‐end tests using commonly available equipment: a CIRS IMRT Thorax phantom, PinPoint ionization chamber, Gafchromic film, and bolus. Four clinical scenarios were evaluated: weight gain and weight loss were simulated by adding and removing bolus, internal target shifts were simulated by editing the CTV during the adaptive workflow to displace it, and changes in gas were simulated by removing and reinserting rods in varying phantom locations. The effect of overriding gas pockets during planning was also assessed.

All point dose measurements agreed within 2.7% of the calculated dose, with one exception: a scenario simulating gas present in the planning CT, not overridden during planning, and dissipating at treatment. Relative film measurements passed gamma analysis (3%/3 mm criteria) for all scenarios. Our process validated the Ethos dose calculation for online adapted treatment plans. Based on our results, we made several recommendations for our clinical adaptive workflow. This commissioning process used commonly available equipment and, therefore, can be applied in other clinics for their respective online adaptive platforms.

## 1 INTRODUCTION

Several online adaptive radiotherapy platforms have been introduced in the past decade, including both MRI‐ and cone‐beam computed tomography (CBCT)‐based systems. These technologies have shown potentially substantial improvements in target coverage and normal tissue toxicity for patients.^1^, ^2^, ^3^, ^4^, ^5^ Due to their increasing commercial availability, and recent promising clinical trial results,^6^, ^7^, ^8^, ^9^ it is highly likely that the number of clinics implementing online adaptive radiotherapy will grow quickly.

Online adaptive radiotherapy platforms require a new clinical workflow and include many new auxiliary systems to complement the standard radiotherapy equipment used for treatment. Several clinics have already successfully implemented online adaptive radiotherapy platforms in the clinic. Although some of these groups have described their clinical adaptive workflow,^4^, ^5^, ^10^, ^11^, ^12^, ^13^ there is limited published experience for commissioning these new adaptive platforms. Hu et al. validated the Acuros dose calculation in the Ethos treatment planning system (TPS) but did not assess the accuracy of the dose calculated during the adaptive workflow.^14^ Commissioning is vital to the safe implementation of new systems and is used to validate the accuracy of the dose calculations involved. Another main goal of commissioning is to understand the strengths and limitations of the system, which can define its appropriate clinical use.

Adaptive platforms inherently include algorithms for auto‐segmentation, auto‐planning, and dose recalculation. Although the results of the auto‐segmentation and auto‐planning can be checked and modified in real time, there are limited methods for assessing the validity of the dose recalculation step before delivering the adaptive treatment plan. Additionally, dose recalculation generally relies on a synthetic CT^15^, ^16^, ^17^ (synCT), which is typically created by deforming the planning CT to the on‐treatment MRI or CBCT image. It is thus vital to evaluate the impact of the synCT on the dose calculation accuracy and the appropriateness of its use for common clinical scenarios.^18^

The purpose of this work is to describe our experience commissioning the Ethos (Varian Medical Systems, Inc., Palo Alto, CA) adaptive radiotherapy platform, with a particular focus on novel tests designed to validate the accuracy of the online dose calculation and delivery with the adaptive workflow. We describe our test process and results for several clinical scenarios, including weight gain and loss, target inter‐fraction displacement, and changes in gas. Additionally, we describe the potential limitations identified within our system and make recommendations for clinical practice. Although these tests were specifically performed on the Ethos, which is a CBCT‐based platform, the same series of tests could be replicated for other online adaptive radiotherapy platforms, including MRI‐based systems. Furthermore, these tests were all performed using common measurement equipment (e.g., rigid phantoms that cannot be physically deformed) so that they could be replicated easily in other clinics. Sharing this experience is essential to provide guidance and recommendations for other groups implementing online adaptive radiotherapy. Without such guidance, implementing this new technology can be challenging and lack standard benchmarks for quality and safety.

## 2 METHODS

**FIGURE 1:**
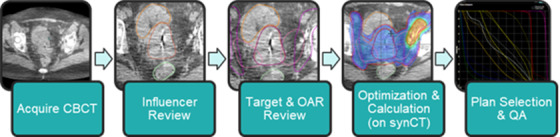
Adaptive workflow on the Varian Ethos cone‐beam computed tomography (CBCT)‐based online adaptive radiotherapy platform. The Ethos creates a synthetic CT (synCT) to calculate the scheduled plan and to optimize and calculate the adapted plan.

At our clinic we installed and commissioned the Varian Ethos v1.0 from June to August 2021. The Ethos adaptive radiotherapy platform^19^ includes an online adaptive workflow utilizing an integrated O‐ring linear accelerator and CBCT imaging system along with novel software systems. In brief, the Ethos adaptive workflow (Figure 1) first auto‐segments a subset of organ‐at‐risk (OAR) structures called influencers using a convolutional neural network on the daily CBCT. The operator edits these as needed and approves them. The approved influencer structures are incorporated in a structure‐guided deformable registration between the planning CT and the daily CBCT acquired on‐treatment. This registration is used to deform the target and other OAR structures onto the CBCT, where they can be edited. The planning CT is also separately deformed to the CBCT using elastic deformable registration to create a synCT for dose calculation. In our study, the PTV is created using an auto‐margin on the edited CTV. The adaptive workflow will then use the user‐approved OARs and target structures to calculate dose for two plans: (1) the *scheduled plan* that is the original plan recalculated on the synCT with dose metrics measured from the edited structures and (2) the *adapted plan* that is optimized and calculated on the synCT also using the edited structures. The operator is then shown the dose and dose metrics from both plans and selects one to use for treatment. Simultaneous to the plan review, the new plans are transmitted to a secondary dose calculation software^20^, ^21^ to repeat and compare the calculation via gamma analysis.^22^ Although this software uses a separate dose calculation algorithm to be independent, it uses the same synCT images for calculation and thus cannot detect an error in its creation or quality.

After installation and acceptance, we performed standard linear accelerator commissioning following the recommendations of MPPG 8.a, including absolute calibration^23^ and standard end‐to‐end phantom test measurements.^24^ To commission the adaptive workflow, particularly the accuracy of the dose calculated on the synCT, we performed a series of end‐to‐end phantom tests for multiple adaptive scenarios. The methodologies used for the end‐to‐end tests are described in the following sections.

### 2.1 Equipment used

**FIGURE 2:**
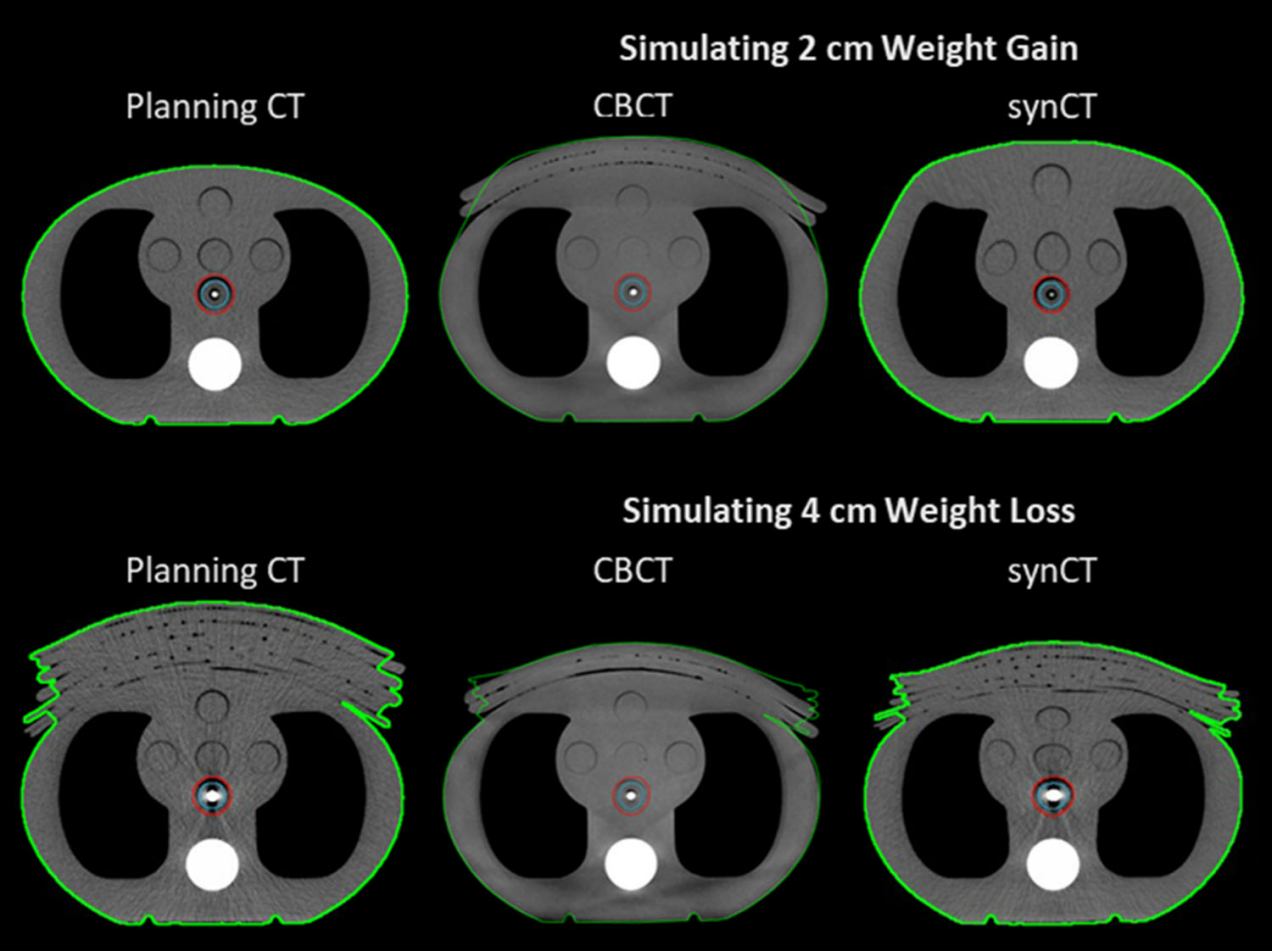
Weight gain and loss scenarios. Axial slice of the planning computed tomography image (CTs) (left column), on‐treatment cone‐beam computed tomography (CBCT) (middle column), and synthetic CT (synCT, right column) for the adaptive end‐to‐end test simulating 2.0 cm of weight gain (top row) and 4.0 cm of weight loss (bottom row). The body contour is shown in green, PTV in red, and CTV in blue.

The phantom used for all adaptive end‐to‐end tests was the heterogeneous CIRS IMRT Thorax phantom (Computerized Imaging Reference Systems, Inc., Norfolk, VA), Figure 2. The phantom has five solid removable cylinders that can be replaced with a cylinder that has a hollow insert space for an ionization chamber. There are additional insert sections, including the lung and spine heterogeneities. Additionally, film can be inserted between slabs of the phantom in the axial plane. For our tests, the delivered dose was measured using a PinPoint ionization chamber Model 31014 (PTW, Freiburg, Germany) for point measurements and Gafchromic EBT‐3 film (Ashland Advanced Materials, Bridgewater, NJ) for planar dose. The chamber readings were converted to dose‐to‐medium using a calibration factor that was derived by delivering a known dose‐to‐medium under reference conditions.^25^ Films were scanned using an Epson Expression 12000XL scanner (Epson America, Inc, Los Alamitos, CA) and converted from optical density to dose using RIT Software v 6.9.64 (Radiological Imaging Technology, Inc., Colorado Springs, CO). The measured planar dose was normalized by the dose measured with the ionization chamber. This dose distribution was compared to the calculated planar dose using gamma analysis with 3% global dose difference and 3‐mm distance‐to‐agreement criteria and threshold >90% points passing.

### 2.2 Adaptive end‐to‐end tests

To validate the dose calculated on synCTs during the adaptive workflow, we designed a commissioning process using standard resources to simulate various common clinical scenarios. Overall, the process involved acquiring a planning CT of the heterogeneous phantom, creating a treatment plan, and then delivering the treatment to the phantom—with systematic adjustments—using the adaptive workflow on the Ethos. Treatment delivery was repeated multiple times with varying modifications to simulate common patient changes seen on‐treatment that can affect the accuracy of the dose delivered: patients gaining and losing weight; target displacement due to internal anatomic changes, such as bladder filling; and the presence of gastrointestinal gas and effect of the gas override protocol. These clinical scenarios are described in detail in Table 1. For each scenario evaluated, the delivered dose was measured with a PinPoint ionization chamber and Gafchromic film and compared to the dose calculated on the synCT (percent difference of measurement from calculation). Each scenario was treated twice: (1) with the scheduled plan selected for treatment (i.e., original plan created on planning CT and calculated on the synCT) and (2) the adapted plan selected for treatment (i.e., plan re‐optimized and calculated on the synCT). Delivered dose was measured for both scheduled and adapted treatments as both are calculated on the synCT.

**TABLE 1:** Adaptive clinical scenarios

**Scenario 1: Weight Gain**	
The heterogeneous phantom was scanned and planned with the ionization chamber inserted	For treatment delivery, Superflab bolus was incrementally added anteriorly to the phantom to simulate weight gain (0.0, 0.5, 1.0, 1.5, and 2.0 cm bolus added)
**Scenario 2: Weight Loss**	
The heterogeneous phantom was re‐scanned and planned with 6.0 cm of bolus anteriorly	For treatment delivery, the phantom was initially setup with 6.0 cm bolus added anteriorly to replicate the setup at the time of CT simulation. Then, bolus was incrementally removed to simulate weight loss (0.0, 0.5, 1.0, 1.5, 2.0, 3.0, and 4.0 cm bolus removed)
**Scenario 3: Target inter‐fraction Displacement**	
The same scan and plan was used as for weight gain (Scenario 1—phantom scanned with no bolus)	For treatment delivery, at the contouring step of the adaptive workflow, the CTV was edited to be displaced 1.0 cm laterally to simulate internal inter‐fraction motion. Separately, a 5.0 cm displacement in the anterior–lateral direction was also tested
**Scenario 4: Changes in Gas**	
*4A: Gas not present at CT*	
The same scan and plan was used as for weight gain (Scenario 1—phantom scanned with no bolus)	For treatment delivery, three of the insert rods were removed to introduce air pockets in the heterogeneous phantom and mimic the appearance of internal gas
*4B: Gas present at CT and not overridden in plan*	
The heterogeneous phantom was re‐scanned with three of the insert rods removed	For treatment delivery, the same rods were removed to simulate the gas still being present at the same location during treatment. Then, the treatment delivery was repeated with these rods reinserted to simulate the gas dissipating. Finally, the treatment delivery was repeated with a different rod removed to simulate gas moving to a new location
*4C: Gas present at CT and overridden in plan*	
The same planning CT from 4B was replanned with the gas overridden to the value of water	The same treatment delivery scenarios described in 4B were repeated (gas still present, gas dissipated, and gas moved)

*Note*: Descriptions of the clinical scenarios evaluated with the adaptive end‐to‐end test protocol, including simulation CT, planning, and treatment delivery. These clinical scenarios were all simulated using a standard heterogeneous phantom with modifications.

Abbreviation: CT, computed tomography.

All plans were created in the Ethos TPS. Dose was calculated for each plan using the Varian Acuros algorithm (v.16.1.0). All phantom plans used 9‐field IMRT, 6x‐FFF beams, and a prescription of 200 cGy per fraction. The plans were created for a centrally located CTV that covered the ionization chamber active volume and extended over the film plane. A 0.5 cm margin was added to the CTV to create the PTV. For a comparison of the calculated dose to measurements, the 3D dose distributions for all plans and resulting dose distributions on the synCTs were transferred to the Eclipse TPS (a necessary step due to current limitations in extracting dose from the Ethos TPS). Additionally, the synCT was qualitatively assessed against the known introduced changes to the phantom for all scenarios, such as any anomalous discrepancies in the body contour from the phantom exterior or deviations from expected location of air pockets.

## 3 RESULTS

### 3.1 Weight gain and weight loss scenarios

**FIGURE 3:**
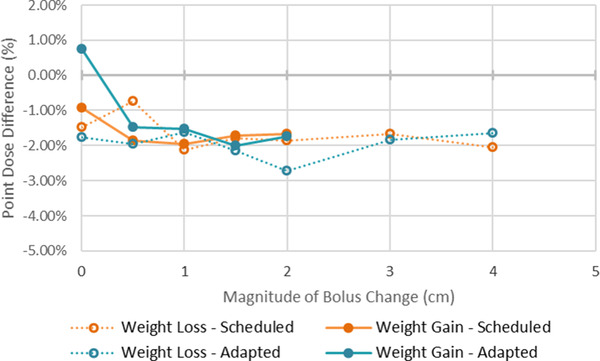
Results from weight gain and loss scenarios. Percent difference in the point dose measured by the ionization chamber and calculated on the synthetic computed tomography (synCT) for the adaptive end‐to‐end tests simulating patient weight loss and gain. Results are shown for when the plan selected for treatment was the scheduled plan and the adapted plan.

**FIGURE 4:**
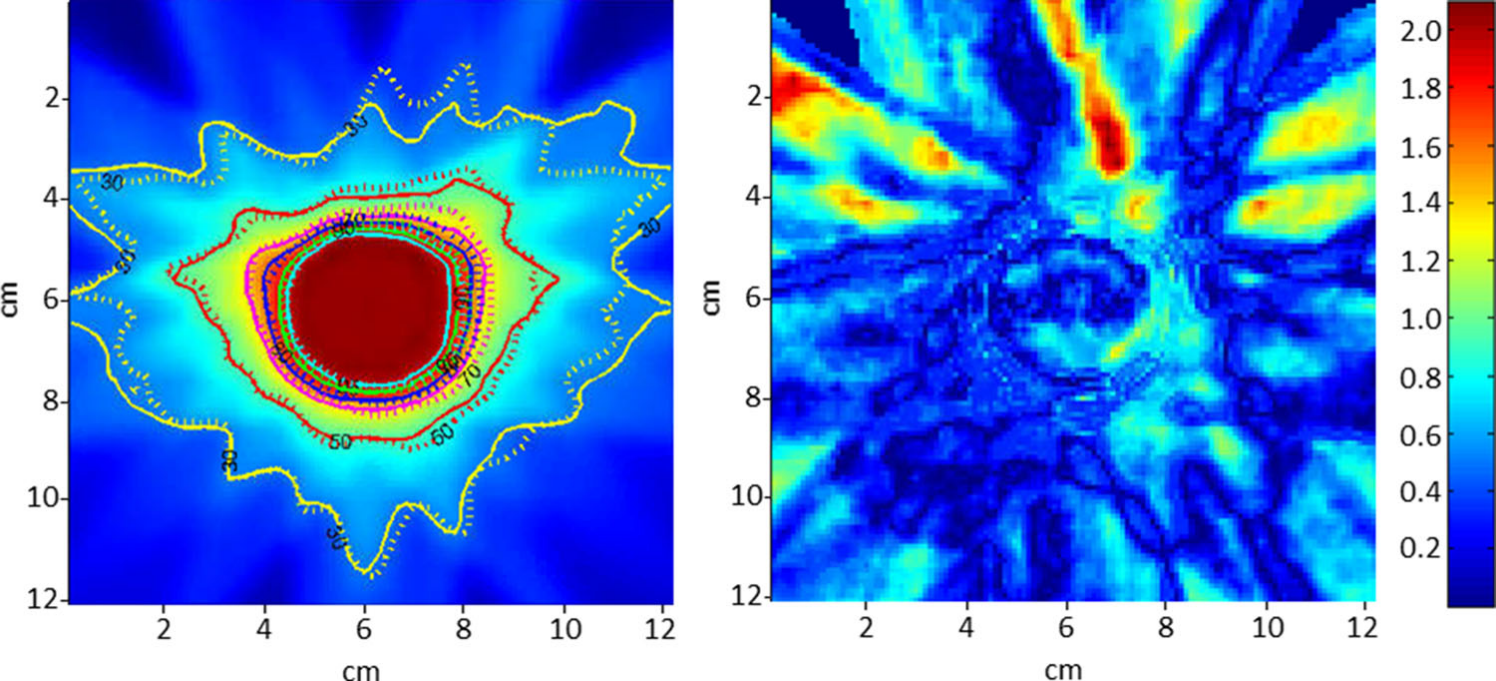
Comparison of the film measurement to the plan for the adapted plan for the 2 cm weight gain scenario. On the left, the plan dose is shown overlaid with isodose lines from the plan (solid lines) and the film (dotted lines). On the right is the result of the gamma analysis (3%/3 mm criteria). In our study, this film was the one with the lowest number of points passing gamma analysis (90.8%). Note that the majority of failing points (gamma > 1.0) are outside of the high dose region and occurred anterior to the target in the same direction that the additional bolus was added.

Examples of the resulting planning CTs, CBCTs, and synCTs of the adaptive end‐to‐end tests for 2.0 cm weight gain and 4.0 cm weight loss are shown in Figure 2. The results of the point dose measurements for the weight gain and weight loss scenarios are shown in Figure 3, where the percent difference in the measurement from the calculation is shown. All ionization chamber measurements agreed well and were within 2.7% of the synCT calculated point dose. The average percent difference for weight gain and weight loss was −1.6% (standard deviation of 0.6%). All measured films for the weight gain and loss tests passed gamma analysis with pass rates >90%. The gamma analysis result with the lowest passing rate is shown in Figure 4 (2.0 cm of weight gain, adapted plan). Across all scenarios, there was no trend in the agreement of measurements with the magnitude of weight gain and loss. Figure 2 also shows representations of the changes in body habitus as handled by the synCT. Body habitus changes on the synCTs versus known bolus thickness changes were assessed for all scenarios in the anterior–posterior direction and were within 1.0 cm of expected values. The body habitus demonstrated reasonable deformations for all weight loss and weight gain scenarios.

### 3.2 Target displacement scenario

**FIGURE 5:**
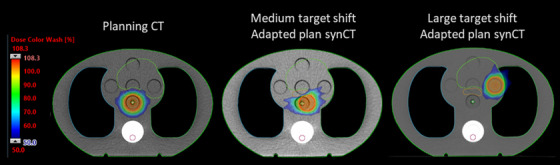
Target displacement scenarios. Axial slice of the planning computed tomography (CT) (left column), synthetic CT (synCT) (middle column) for the adaptive end‐to‐end test simulating a 1.0 cm internal shift of the target (middle column), and synCT for the adaptive end‐to‐end test simulating large 5.0 cm internal shift of the target (right column)

The results of the two separate scenarios simulating internal shifts of the CTV (1.0 cm in the lateral shift and a larger shift of 5.0 cm in the anterior–lateral direction) are shown in Figure 5. For the medium 1.0 cm shift, the measured point dose was within −2.0% and −1.4% of the calculated dose for the scheduled and adapted treatment, respectively. Both dose distributions showed high agreement with gamma pass rates of 99.0% or above. When a larger shift of 5.0 cm in the anterior–lateral direction was applied, we found that this large displacement resulted in the scheduled plan to be undeliverable due to extensive dose deviations (a safety feature of the Ethos system). However, the adapted plan could accommodate such large shifts. The measurements for the adapted plan with this large displacement agreed well with calculation (point dose difference of 0.1% and gamma pass rate of 97.2%).

### 3.3 Changes in gas scenario and effect of gas override protocol

**FIGURE 6:**
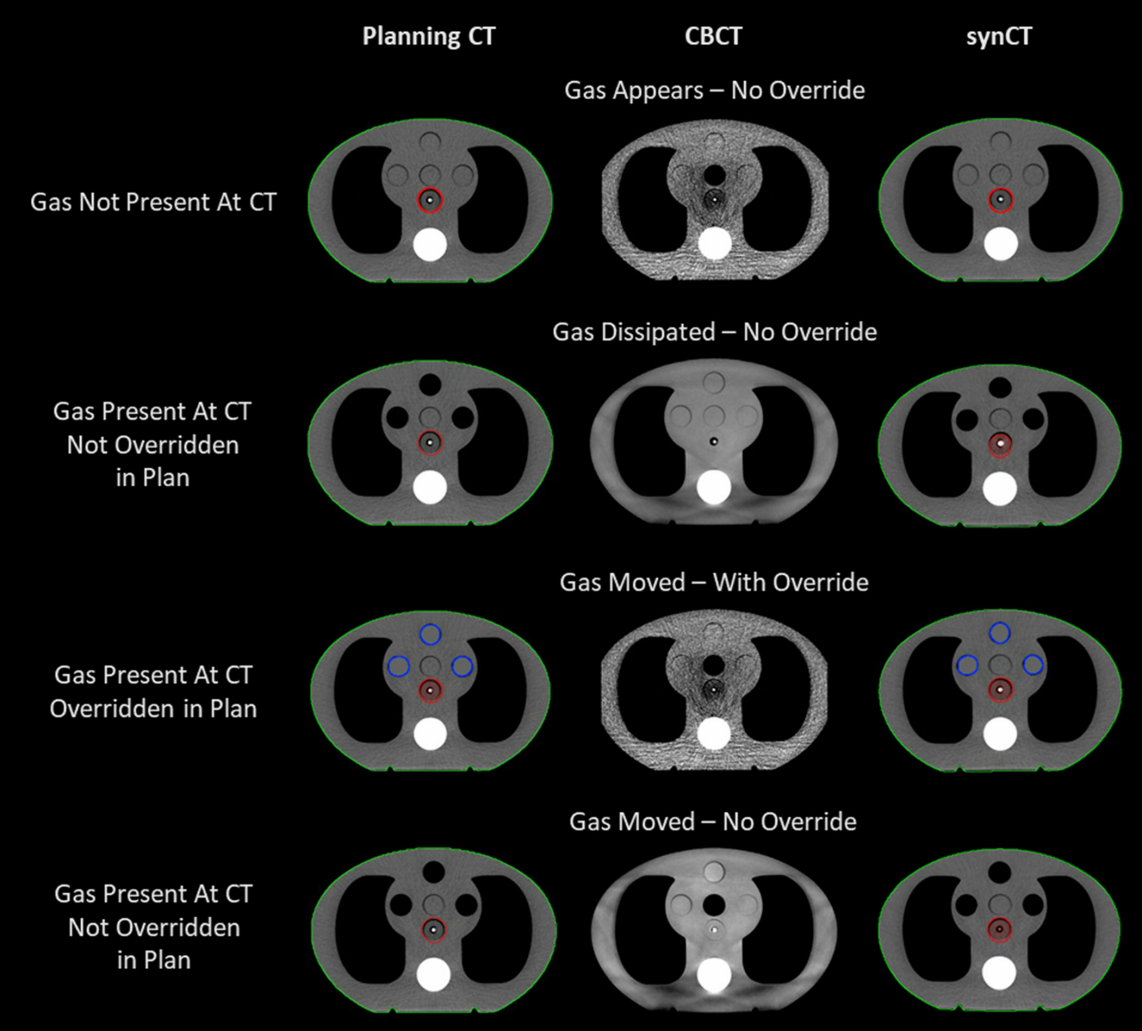
Changes in gas with and without override. Axial slice of the planning computed tomographies (CTs) (left column), on‐treatment cone‐beam computed tomography (CBCT) (middle column), and synthetic CT (synCT, right column) for the adaptive end‐to‐end tests simulating changes in gas. The top row represents when gas was not present at CT, but appeared on‐treatment. The second row represents when gas was present at CT, not overridden in the planning image, but dissipated on‐treatment. The third row represents when gas was present at CT, was overridden, and moved to a new location on‐treatment. The bottom row represents when gas was present at CT, was not overridden, and moved to a new location on‐treatment. The body contour is shown in green, PTV in red, and gas override in blue.

**FIGURE 7:**
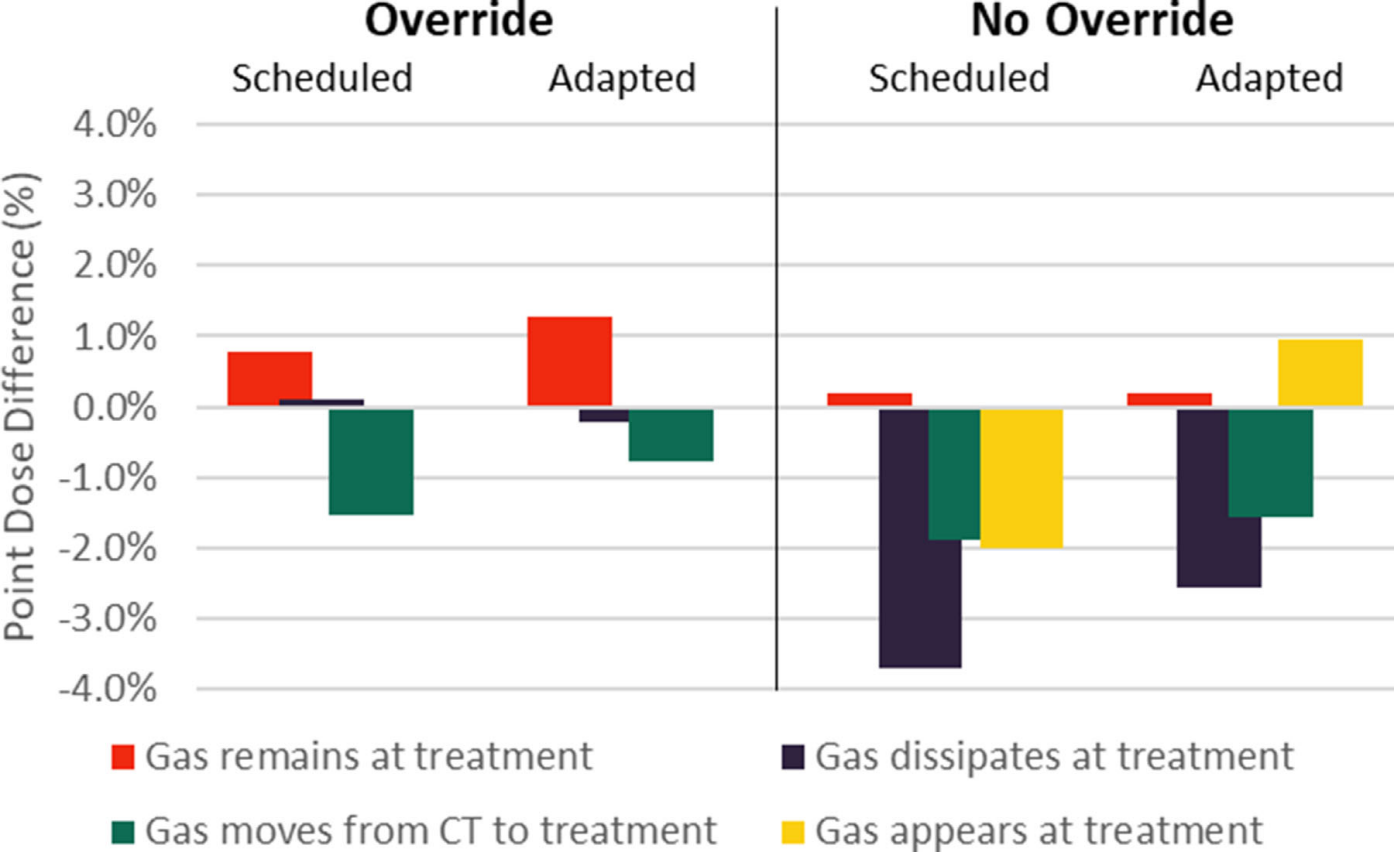
Effect of gas override protocol on calculated dose accuracy. Percent difference in the point dose measured by the ionization chamber and calculated on the synthetic computed tomography (CT) for the adaptive end‐to‐end tests simulating changes in gastrointestinal gas. When gas was present at CT simulation and overridden in the initial plan, treatment was delivered with (1) the gas remaining unchanged, (2) gas dissipating, and (3) gas moving to a new location. These tests were repeated with a new plan in which gas was not overridden. Finally, a test was performed where no gas was present at CT simulation and then appeared at treatment delivery. Results are shown for when the plan selected for treatment was either the scheduled plan or the adapted plan.

Examples of the resulting CTs, CBCTs, and synCTs of the adaptive end‐to‐end tests for changes in gas are shown in Figure 6. The results of the point dose measurements are shown in Figure 7; the average percent difference for all scenarios was −0.8% (standard deviation of 1.5%). The measurements agreed well with dose calculated on the synCT for all simulated changes in gas scenarios with one exception: the scenario in which gas was present at simulation and not overridden for planning (Scenario 4B), and then had dissipated at treatment. The difference in calculated and measured point dose was −3.7% and −2.6% for scheduled and adapted treatments for this scenario. All gamma pass rates showed good agreement and were greater than 94%.

When qualitatively assessing the CT values in the synCT, we observed that density overrides included for planning were propagated through to the synCT, as demonstrated in Figure 6 in the bottom row. Therefore, if bowel contrast or gas were overridden for planning, these overrides would be included for calculating dose in the adaptive workflow. Similarly, if no gas was present at the time of simulation, there will be no gas in the synCT regardless of how much gas is present at the time of treatment. We also observed that large changes in gas, such as shown in the bottom row of Figure 6, were not deformed accurately, as represented in the synCT.

## 4 DISCUSSION

Online adaptive radiotherapy platforms present a unique challenge for commissioning as guidance is lacking and specialized adaptive equipment, such as deformable phantoms, are exceedingly rare. In this work, we designed and implemented a series of novel end‐to‐end tests using standard phantoms to assess the accuracy of the online dose calculation and to understand the limitations of the Ethos adaptive radiotherapy platform. These tests were designed to make users aware of the magnitude of uncertainty that will be introduced by common clinical scenarios. Additionally, these tests were designed to be flexible and accessible to many clinics as they use common resources found in a radiation therapy department such as a heterogeneous phantom and bolus. Lastly, the created plans and results can be used as baselines to reevaluate the system after software upgrades to the adaptive workflow.

Our results validated the online dose calculation on the synCT as part of the adaptive workflow of the Ethos platform. We generally saw robust results for dose calculation, even with charges as large as 4.0 cm in patient anatomy. Some of the largest deviations in measurement were at moderate changes in body habitus (e.g., −2.0 cm), although the point measurement results were within two standard deviations of the mean and largely still in the acceptable range. Additionally, no trend in measurements was observed, suggesting that sources of error were not systematically tied to changes in body habitus. The largest deviation in computed and measured point doses occurred when large gas pockets were not overridden in the initial treatment plan and then dissipated at treatment, where they were as large as 3.7%. All films showed high agreement with greater than 90% of points passing for all plans. The largest discrepancy occurred for the adapted plan in the 2.0 cm weight gain scenario, Figure 4. For this case, the failing regions were located outside the target in the anterior portion of the phantom, which may be due to discrepancies in the deformation of the lungs.

Another result of this study was a better understanding of the limitations, strengths, and key functionality of the online adaptive workflow with the Ethos platform. Namely, we gained an expectation of the results with a range of common changes in anatomy. We found this validation to be especially important for the current version of Ethos as the synCT cannot be verified visually at the time of treatment. One important result of this study was that the body habitus of the synCT was assessed and verified to match the actual body habitus changes introduced. We have found that using the contours of the body and of the high‐density structures, which includes the bony anatomy, are a good surrogate for assessing if the synCT is appropriate to the anatomy during the online workflow. Key functionality we observed through this testing was that density overrides in the initial treatment plan will be propagated to the synCT and be used for online dose calculation. We also observed that the adaptive workflow could accurately accommodate large internal changes in anatomy, including shifts of the CTV of 5.0 cm, a benefit of the adaptive treatment approach.

An important part of commissioning is gaining an understanding of the performance of a local system and how it functions under various clinical conditions. For adaptive radiotherapy, the commissioning process should provide guidelines and thresholds for under what conditions the online dose calculation may deviate from reality and, thus, offline re‐simulation and planning should be recommended. Toward this goal, the tests presented in this work were designed to cover various changes in patient anatomy commonly encountered over a full course of radiation therapy. These tests led us to make the following key clinical policies for our adaptive workflow:

1. The accuracy of the body contour and high‐density structure contour relative to the CBCT anatomy should be evaluated for all adaptive fractions to identify issues with the generation of the synCT. For large systematic changes in body size (2.0 cm or more), re‐simulation with offline replanning remains an option.
2. Any materials present at the time of CT simulation that are not expected to be present at treatment, such as bowel contrast, must be overridden in the planning CT.
3. Other regions in the scan with a density that may vary over a course of radiotherapy, such as large gas bubbles, should be overridden with care and monitored during treatment. Ideally any such overrides should be communicated to the team members that will be evaluating the daily adapted plans so that they are aware of regions where the daily adapted or scheduled dose may be less accurate and can take this information into consideration when selecting a plan.

The beam model and calculation algorithm have been previously verified for the Ethos TPS for initial treatment planning on the simulation CT.^14^ However, this does not consider the impact of the synCT on the dose calculation in the adaptive workflow. A few groups have assessed the end‐to‐end dose accuracy for various MR‐based adaptive platforms using specialized phantoms, some constructed in‐house.^26^, ^27^, ^28^, ^29^ Although this approach is useful for providing a generalized idea of the accuracy of adaptation using a specific platform, it cannot be replicated by clinics commissioning their own systems without the acquisition or construction of specialized equipment, such as deformable phantoms. Additionally, all those publications focused on MR‐based online adaptive radiotherapy.

We performed our tests on the Ethos system that does not allow visualization or hand editing of the synCT used for dose calculation. However, the MRIdian adaptive platform (ViewRay Inc., Oakwood Village, OH) allows the synCT to be visualized and edited as necessary (e.g., for large air pockets), which we anticipate would decrease the point dose differences we observed in our air‐override tests. It would still be valuable with that system to perform a series of end‐to‐end tests, like those described here, to validate that the platform is performing these steps with high accuracy.

A strength of the proposed commissioning process was that it used commonly available equipment, including phantoms, ionization chambers, and film. Although we relied specifically on the CIRS IMRT Thorax phantom for this work, the principles behind these tests could be completed with any phantom capable of introducing heterogeneity changes and with space for measurement equipment. Additionally, this process is general enough that it could apply to other adaptive radiotherapy platforms, as long as the equipment is compatible (e.g., an MRI compatible phantom for an MRI‐based adaptive platform). Although our study focused on the technical aspects of safely commissioning adaptive radiotherapy, clinics should also give careful consideration to procedures and guidelines for contouring and evaluating adaptive plans. A recent paper by Kim et al. provides some guidance from physicians for ART prescriptions and on‐treatment decision‐making,^30^ whereas a publication by Shepherd et al. details an approach to training advanced adapters and implementing an adaptive workflow.^31^

A limitation of this work is that this series of tests is non‐exhaustive and could be expanded to include other scenarios that clinics may encounter with online adaptation. Most of these tests were designed considering the adaptation of pelvic sites, such as prostate and cervical cancer. As such, the scenarios simulated changes are regularly seen for pelvic anatomy. Additional tests could be considered, which would address common clinical scenarios experienced in other anatomical regions such as respiratory motion, or multiple scenarios could be combined and evaluated (e.g., simultaneous weight loss and changes in gas), as this commonly occurs clinically.^32^ Furthermore, if clinics do have access to phantoms that can include known deformation, these would also be beneficial for validating online adaptive workflows and understanding limitations of adaptive platforms. However, the simple tests described in this study were essential to our understanding of the limitations and functionality of our adaptive platform. Finally, we used 3% and 3 mm criteria for our gamma analysis, which is our standard clinical practice for patient‐specific IMRT QA. However, other sites may use tighter criteria (e.g., 2% and 2 mm) particularly during commissioning. This would likely result in lower pass rates in the previous tests and thus potentially stricter recommendations for patient suitability for adaptation. More uncertainties are present in adaptive planning than standard IMRT QA, as highlighted in the scenarios we analyzed earlier; thus, we feel using our conventional thresholds from IMRT QA is appropriately strict to evaluate the safety of this technique without unduly limiting its clinical usability.

## 5 CONCLUSION

In this study, we presented a novel process for commissioning the adaptive workflow for an online adaptive radiotherapy platform. We applied this methodology to commission the Ethos adaptive platform in our clinic, validated the online dose calculation for online adaptation, and gained an understanding of the strengths and limitations of the adaptive workflow. This commissioning process used commonly available equipment and, therefore, can be applied in other clinics implementing online adaptive platforms.

## AUTHOR CONTRIBUTIONS

Kelly Kisling: conceptualization, project oversight, investigation, methodology, data analysis, and manuscript writing; Timothy D. Keiper: investigation, data analysis, reviewing, and editing; Daniela Branco: investigation, data analysis, reviewing, and editing; Grace Gwe‐Ya Kim: methodology, data analysis, reviewing, and editing; Kevin L Moore: conceptualization, methodology, data analysis, reviewing, and editing; Xenia Ray: conceptualization, project oversight, investigation, methodology, data analysis, manuscript writing.

## CONFLICT OF INTEREST

Kelly Kisling acknowledges honoraria and travel fees from Varian Medical Systems. Xenia Ray acknowledges honoraria and a research agreement with Varian Medical Systems. Grace Gwe‐Ya Kim acknowledges honoraria and consulting fees from Varian Medical Systems. Kevin L Moore acknowledges honoraria, travel, and consulting fees from Varian Medical Systems.

## Data Availability

The data that support the findings of this study are available from the corresponding author upon reasonable request.
